# A comparative analysis of nanocoated expanded polystyrene for sustainable infrastructure applications

**DOI:** 10.1038/s41598-025-01257-y

**Published:** 2025-05-17

**Authors:** Moamen A. Shekib, Emad S. Bakhoum, Mohamed M. Omran, Ahmed M. Ahmed, Irene Samy Fahim, Sherif S. AbdelSalam

**Affiliations:** 1 SESC, Smart Engineering Research Centre, Nile University, Giza, Egypt; 2 Civil & Construction Engineering, School of Engineering & Applied Science, Nile University, Giza, Egypt; 3 Industrial Engineering, School of Engineering & Applied Science, Nile University, Giza, Egypt; 4 Civil Engineering Department, National Research Centre, Giza, Egypt; 5 Department of Civil Engineering, Faculty of Engineering-Mataria, Helwan University, Cairo, Egypt

**Keywords:** Roadway embankment, EPS geofoam, Geomembrane, Nanocoating, Life cycle assessment, Life cycle cost, Environmental impact, Environmental sciences, Engineering

## Abstract

Engineers increasingly face challenges due to the demand for sustainable infrastructure; thus, this research evaluates different road embankment techniques based on sustainability. It compares the environmental impacts and economic feasibility of three road embankment techniques: using traditional soil backfilling, using expanded polystyrene (EPS) geofoam blocks with geomembrane protection, and using EPS blocks coated with a newly developed nanocoating material. Life Cycle Assessment (LCA) and Life Cycle Costing (LCC) methods are employed to measure the potential environmental loads and economic viability of a real-world twin-bay tunnel project. LCA has been performed with SimaPro software and using ReCiPe endpoint method. The findings indicate that EPS coated with nanocoating material has the lowest environmental impact and cost, with LCA single score of 1.06 MPt and cost reductions of about 14.5% and 27.48% compared to EPS with geomembrane and soil backfilling, respectively. Traditional soil backfilling has the highest environmental impact with a single score of 2.52 MPt. In comparison, EPS with geomembrane has a single score of 2.477 MPt offering a 15.17% cost reduction compared to soil backfilling. Sensitivity analysis concludes that the required amount of reinforced concrete for the tunnel significantly impacts the results. The outcomes support sustainable decision-making for infrastructure solutions.

**Supplementary Information:**

The online version contains supplementary material available at 10.1038/s41598-025-01257-y.

## Introduction

Due to the importance of roadway construction to the nation’s economic development, modern techniques can change the concept of analysis and selection of alternatives for embankments in such projects. Selecting the optimum alternatives must meet the sustainable criteria, including economic feasibility, minimizing the environmental impact, and saving construction time by speeding up the embankment construction process. Geosynthetics provides several embankment alternatives, including expanded polystyrene (EPS) geofoam blocks and geomembranes. These products are produced under control, primarily from polymeric materials, for use in contact with soil, rock, and/or other materials such as concrete^1^. Ensuring the sustainability of these geosynthetic materials is essential to be adopted in civil engineering applications while achieving the Sustainable Development Goals (SDGs)^2^.

EPS geofoam has been successfully used for many years in several infrastructure projects such as the construction of highway and railway embankments, bridge abutments, landscape, and other challenging applications that require lightweight backfilling. EPS blocks are made of thermoplastic materials and are extremely lightweight, hence reducing lateral or bearing stresses. It can be used in varied sizes, shapes, and densities to fit project requirements. Moreover, it can be easily handled without special equipment and installed in various weather conditions. These characteristics make EPS geofoam blocks a reasonable alternative to traditional construction practices, which can provide safe and economic solutions when properly designed, especially when substructures are exposed to static and dynamic loads^3–6^. However, proper protection from ultraviolet rays, chemicals, hydrocarbons, heat, and degradation by insects and termites is required for EPS blocks. To mitigate these problems, the most common way is to incorporate a protective layer around the EPS blocks using high-density polyethylene (HDPE) geomembrane^7,8^. HDPE geomembranes are widely used in many civil engineering applications, but they have some environmental limitations^2^. Therefore, researchers are currently working on finding an eco-friendly organic alternative for geomembrane that can meet the SDGs and enhance EPS geofoam mechanical properties.

A new nanocoating material was developed based on nanocellulose extracted from agricultural waste as a sustainable alternative for EPS protection, and it can also enhance the overall strength and durability of EPS. Nanocellulose is an organic, renewable nanomaterial with favorable characteristics in many fields and applications due to its remarkable chemical and physical properties. Sugarcane bagasse, a fibrous waste material left after crushing sugarcane for juice extraction, is selected to be used as a raw material to extract nanocellulose coating. The developed nanocoating was based on bagasse nanocellulose mixed with zinc oxide (ZnO) to enhance durability, and polyvinyl acetate (PVA) as a binder^9^. This EPS nanocoating was enhanced by Omran et al., after adding and testing another layer of solvent-free polyurethane to resist hydrocarbons and chemicals. However, these studies did not assess the environmental impact of using the newly developed EPS nanocoating^10^.

Life Cycle Assessment (LCA) has played a crucial role in assessing the environmental impact of infrastructure materials and methods. It systematically assesses products and processes across their life cycle, aiding in informed decision-making for sustainable development. The methodology, governed by ISO 14,044 and 14,040 standards, ensures reliable metrics for water use, energy consumption, and CO_2_ emissions^11,12^. Previous researchers highlighted the sustainability of using EPS geofoam blocks in road construction. EPS showed potential for reducing environmental burdens and enhancing ecosystem services compared to traditional methods. It can reduce the amount of required reinforced concrete (RC) in substructures. Highlighting LCA’s role in eco-friendly design and geosynthetic materials, using polyurethane foam in railway substructures significantly reduced water use by 82% and CO_2_ emissions by 5%^12–14^. The life cycle of EPS identified the global warming potential as the most significant environmental impact during manufacturing. The production of EPS consumes about 0.83 to 1.67 kWh/kg of energy. It was recommended to improve the EPS recycling processes to reduce emissions and foster sustainable disposal methods^15,16^.

Life Cycle Costing (LCC) is a methodology used to estimate total costs over a project’s lifespan, covering initial construction to end-of-life expenses. Despite its benefits in comparing alternatives in infrastructure projects, industrial adoption of LCC methods is slow, with practitioners often modifying techniques for specific situations^17^. LCC’s role is important in life cycle management by offering economic insights, which are crucial for addressing the challenges and opportunities of adopting LCC and aligning it with sustainability objectives^18^. Previous studies concluded that the lightweight EPS bead mixture and EPS block methods had high LCC due to raw material costs, while recycling materials helped reduce expenses. The foamed waste glass and conventional cut-and-fill methods demonstrated relatively low LCC^13,19^. LCA and LCC methods were used to analyze the climate impact and cost of railway embankments during production and construction. It was concluded that stabilized sandy fill with blast furnace slag cement had a lower climate impact and cost compared to crushed bedrock. Cement production significantly influenced the climate impact of sandy fill, while material transport and soil excavation were key contributors for crushed bedrock^20^.

Embankment materials encompass a wide range, with extensive research covering options such as recycled plastics, tire chips, volcanic ash, and rice husk, each offering distinct properties. Sugarcane bagasse was used as an agricultural waste for material development and waste management. Previous studies concluded that incorporating bagasse ash into limestone calcined clay cement to replace ordinary Portland cement improved compressive strength, setting time, durability, compaction, and reduced swell potential of black cotton soil. The technical feasibility and cost-effectiveness of using this waste for soil stabilization, addressing climate change challenges, and promoting sustainable practices were highlighted^21–23^.

Most of the studies conducted on the use of EPS geofoam in engineering applications provided valuable insights into the behavior and material performance, primarily focusing on its mechanical properties. Few have examined the environmental impact of the EPS production, use, and disposal phases. Despite EPS geofoam’s established engineering benefits in applications such as roadway embankments, detailed comparisons between the use of traditional soil backfill materials and modern alternatives, like the use of EPS blocks and geomembrane protection, from both environmental and economic perspectives, are lacking. Additionally, the newly developed nanocoating material for EPS protection has not yet been investigated environmentally or economically. This gap represents a significant limitation in understanding the broader implications of using EPS geofoam with geomembrane protection, or nanocoating protection, in infrastructure embankment applications. Consequently, the research question arises: “What is the environmental and economic feasibility of using EPS geofoam blocks, protected with HDPE geomembranes or coated with the newly developed nanocoating material, in roadway embankment applications compared with the traditional soil backfill method?”

This research aims to assess and compare the environmental impacts and economic feasibility of three alternatives for road embankment application: soil backfilling using dense sand and gravel (as a traditional approach), EPS geofoam coated with HDPE geomembrane (as a modern technique), and EPS geofoam coated with nanocoating material (as a new advanced approach). The outcomes provide a framework for evaluating sustainable infrastructure solutions and supporting decision-making in civil engineering projects. Traditional soil backfilling is anticipated to have the lowest environmental impacts and costs due to its natural properties and low initial expenses. However, the question remains: do structural design and other parameters influence this expectation? The findings of this study are limited to roadway embankment projects. They are influenced by factors such as geographic region, material availability, material specifications, costs (including materials, labor, and machinery), and local environmental regulations.

## Methodological framework

**Fig. 1:**
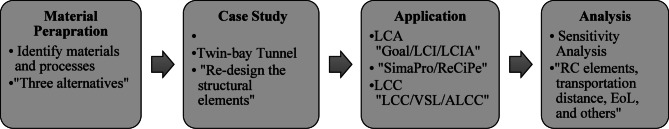
The sequence of the methodological research steps.

The LCA method was used to quantify the potential environmental loads during the alternative’s life cycle stages, from production to end-of-life (cradle to grave). Additionally, LCC was employed to estimate the costs of various alternatives and quantify all associated expenses throughout their service life. Both LCA and LCC were utilized in a case study involving an embankment over a twin-bay tunnel as a real-world application, considering the structural design of the tunnel based on each embankment alternative. The methodological research steps can be summarized in Fig. 1 as follows:

- Identify materials and processes required for the suggested embankment alternatives from extraction to the end-of-life: (1) traditional soil backfilling using dense sand and gravel, (2) EPS geofoam with HDPE geomembrane, and (3) EPS geofoam with nanocoating.
- Select a real-life case study, “The Kamena Vourla twin-bay tunnel in Greece” to assess and compare the three alternatives after re-designing the structural elements of the twin-bay tunnel of the case study to quantify the changes in the amount of reinforced concrete needed for each alternative.
- Apply LCA through its standard steps: defining the study’s goal and scope, collecting the life cycle inventory (LCI), conducting the life cycle impact assessment (LCIA), and interpreting and analyzing the results using SimaPro software and ReCiPe Endpoint (global) 2016 method.
- Apply LCC for the three alternatives, considering their lifetime costs to determine economic feasibility. The health-related costs associated with each alternative, as assessed by LCA, are considered using the value of statistical life (VSL) method.
- Conduct a sensitivity analysis to identify which variables most significantly affect the results and how different scenarios or assumptions can influence the outcomes.

## Materials and processes

The study involves four main materials used for the roadway embankment: soil backfill, EPS geofoam blocks, HDPE geomembrane, and a new nano coating material made from nanocellulose. In addition, concrete and reinforcement steel are utilized as structural materials for the twin-bay tunnel case study. The specifications and processes related to these materials’ extraction, manufacturing, construction, and end-of-life stages are identified.

First, the soil backfill consists of a 1:1 mixture of dense sand and gravel, with a bulk density of 20.42 kN/m³. It is typically applied in 25–30 cm thickness layers, each compacted using mechanical rollers and water to ensure reaching an acceptable compaction effort and provide optimal stability.

Second, the EPS geofoam uses blocks measuring 1 m x 1 m, with a density of 25 kg/m^3^. It offers advantages such as being lightweight, having high compressive strength, low density, excellent thermal insulation properties, and being easy to handle and install^24^.

Third, the HDPE geomembrane with a density of 930 kg/m^3^ is used to protect the EPS blocks. It provides an effective barrier against moisture and environmental degradation. The geomembrane used has a thickness of 2 mm and typical roll dimensions of 4 m in width and 10 m in length. HDPE geomembranes are known for their excellent chemical resistance, UV stability, and mechanical durability. The installation process involves unrolling the geomembrane over a solid substrate, ensuring proper overlap between adjacent sheets, and using thermal welding techniques to create watertight seams^25^.

The last material used in this study is the innovative nanocoating developed by Adel et al. and Omran et al., used to enhance EPS properties and provide an alternative protective layer for the geomembrane. This nanocoating contains advanced components like nanocellulose from sugarcane bagasse (SCB), zinc oxide, PVA, and elastic polyurethane in epoxy form as a protective layer over the nanocoating^9,10^. Supplementary Figure (A) presents the preparation of nano-coating involving a multi-step treatment process (Appendix A – Supplementary data). The treatment process was summarized by^9^, which starts with washing the SCB with distilled water and drying at 80 °C for 24 h. For alkaline hydrolysis, the bagasse is treated at room temperature with a 15% NaOH solution for 4 h, then repeatedly washed with distilled water to remove the alkali. The fibers are dried at 80 °C for 24 h. The biomass is then soaked in a 1 mol HCl solution at 80 °C for 4 h. A second alkaline hydrolysis involves treating the material with a 2% NaOH solution for 4 h at 80 °C, followed by washing with distilled water and drying at 70 °C for 1 h. Ultrasonication is performed at 40% amplitude for 30 min, with cellulose mixed at a 4:1 water ratio^9^. The SCB nanocellulose is applied on the EPS block and left to cure for 6 h. The average thickness of the nano SCB layers after application should be approximately 0.7 mm. Then a secondary layer of polyurethane is applied as recommended by^10^. Polyurethane is applied twice in two perpendicular directions to ensure full and uniform coverage, with an approximate thickness of 0.1 mm. The polyurethane coating reaches the final setting time after 24 h and achieves full hardness after 7 days.

## Case study

### Description

**Fig. 2:**
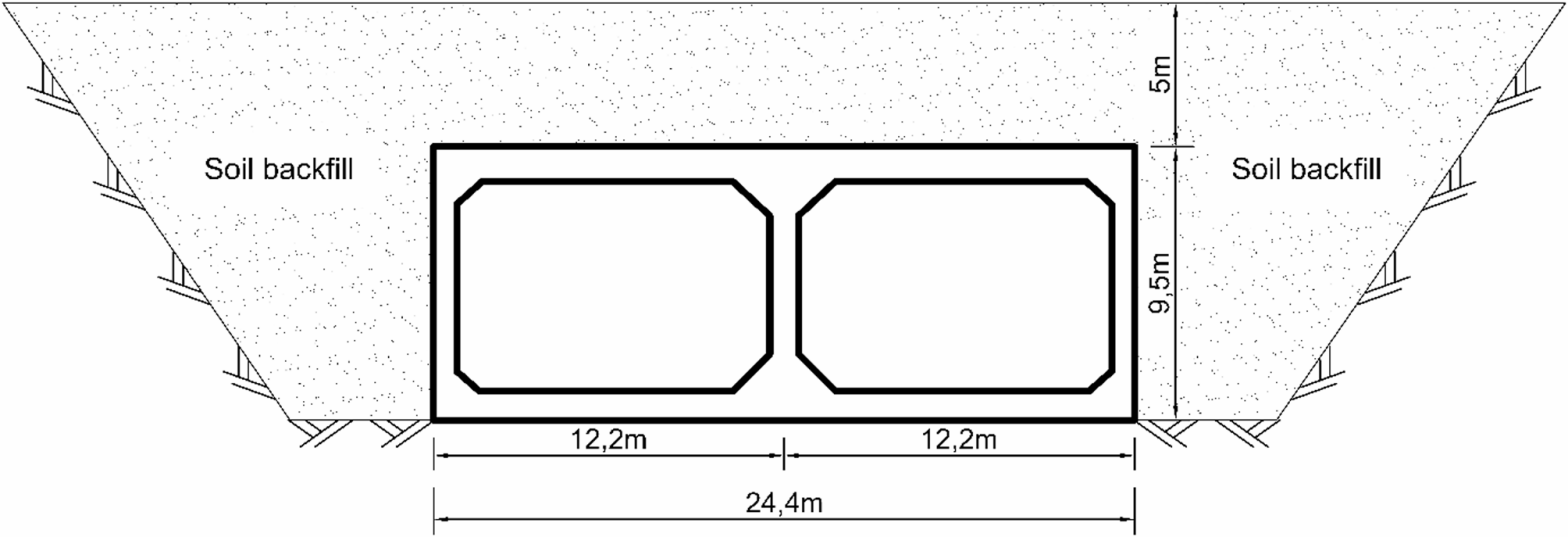
Cross-section dimensions of “Kamena Vourla” tunnel.

Kamena Vourla Tunnel Bay, located in central Greece, is the case study for this research. It is a part of a highway that extends 550 km linking southern and northern Greece. The twin-bay tunnel extends 400 m of the road and comprises two box sections with an overall width of 24.4 m and a height of 9.5 m. The original tunnel was covered with a 5 m depth of soil backfilling over its deepest profile. Figure 2 illustrates the cross-section dimensions of the tunnel covered with soil backfill.

### Structural design of the tunnel

Researchers have explored the behavior of underground structures when backfilled with EPS instead of traditional soil, considering various parameters. Anastasopoulos et al.^26^ studied the performance of the underground twin-bay tunnel Kamena Vourla. Separately, AbdelSalam et al. analyzed the seismic impact on the foundation soil for the same tunnel under two conditions: full soil backfilling and a combination of EPS and soil backfilling.

In this study, the underground twin-bay tunnel “Kamena Vourla” was redesigned to compare the required amount of concrete and reinforcement steel for the tunnel using the three different backfilling methods. The tunnel was designed according to the ECP 203–2018^27^ utilizing normal-strength concrete with a compressive strength of 30 MPa and moderate workability with a 100 mm slump. The reinforcement used was non-welded steel (B400C-R) with a yield strength of 400 MPa. Loads in the design included live loads over the backfilling layer equal to 0.01 kN/m^2^ and live loads inside the tunnel equal to 0.084 kN/m^2^. Table 1 summarizes the soil properties under the foundation of the tunnel and for backfilling.

**Table 1:** Soil and backfilling soil properties for the tunnel.

Parameter	Soil properties Mohr-Coulomb	
	Soil profiles under the foundation level	Backfill soil
Bulk density “γ_b_” (kN/m^3^)	17	20.42
Saturated density “γ_sat_” (kN/m^3^)	19	31.42
Elastic modulus “E_0_” (kPa)	35 × 10^3^	65 × 10^3^
Cohesion “c” (kPa)	5	2
Friction angle “ϕ”	35	46
Poisson’s ratio “ʋ”	0.3	0.3

### Structural design results and analysis

**Fig. 3:**
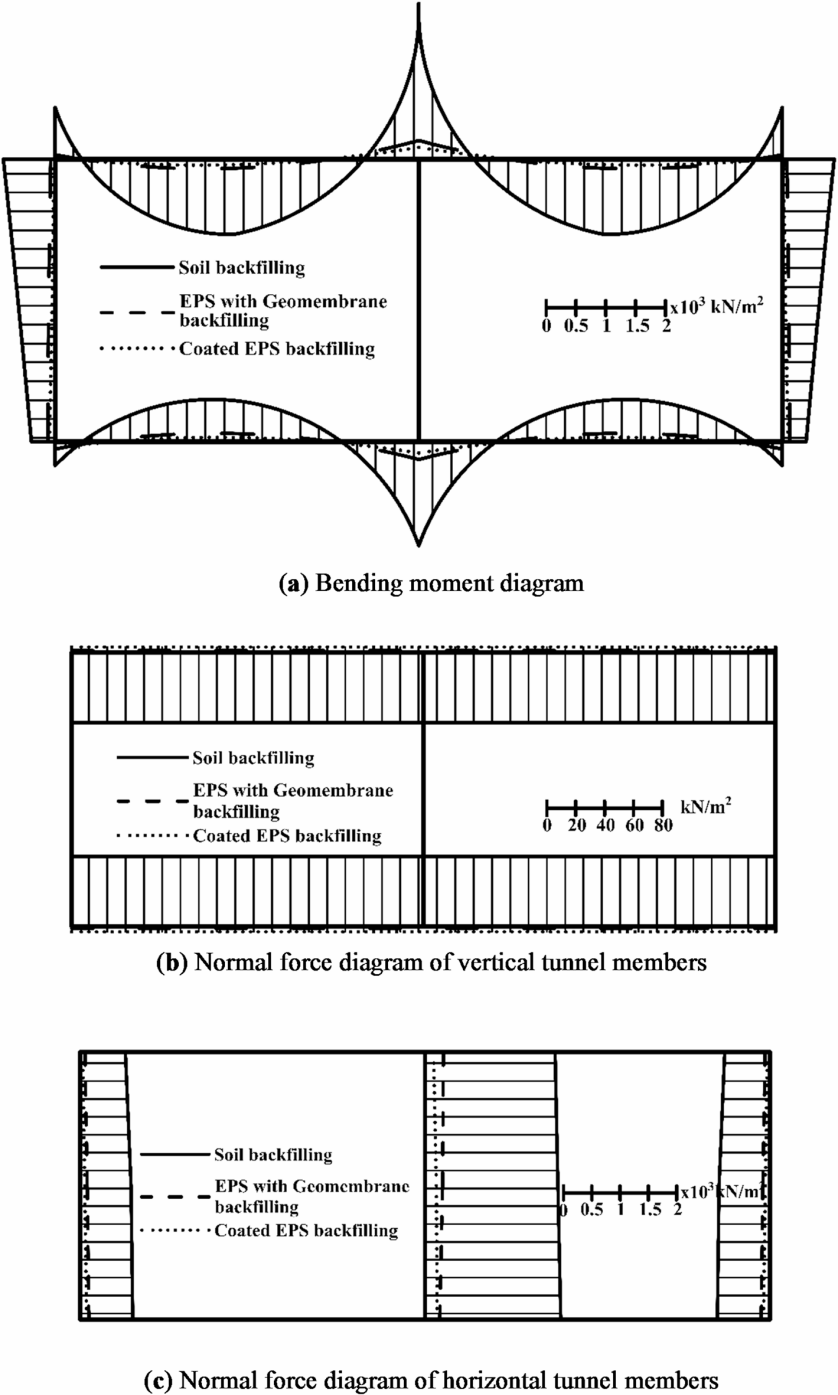
Comparison between the structural analysis outcome of the tunnel.

The structural analysis of the three cases reveals a substantial reduction in bending moment by 88.3%, and a decrease in normal force by 81.6%, at the critical sections when using EPS with HDPE geomembrane. When nanocoated EPS is used as an alternative to soil backfilling, there is a 93% reduction in bending moment and an 84% decrease in normal force. This difference is due to the relatively lighter weight of the nanocoating compared with the HDPE. Figure 3 illustrates the comprehensive straining actions of the tunnel. The design of critical tunnel members leads to a significant reduction in the quantities of concrete and reinforcement needed. Where the quantities of required concrete for the soil backfilling, EPS with HDPE geomembrane, and EPS with nanocoating are 29,600, 11,480, and 8680 m^3^ respectively. The quantity of concrete decreased by 61% when using EPS with HDPE geomembrane and by 71% when using EPS with nanocoating instead of soil backfilling. The quantity of concrete needed when using nanocoated EPS was 76% of that required for EPS with HDPE. In addition, the required quantities for steel are 4525, 1942, and 1743 ton respectively. The amount needed with EPS and HDPE was 43% of that required for soil backfilling while using nanocoated EPS required 90% of the reinforcement needed for EPS with HDPE. This reduction in materials directly impacts the environmental footprint and cost and indirectly influences the structure’s behavior and durability.

Compared to earlier research by Anastasopoulos et al.^26^, and AbdelSalam et al.^28^, their findings indicated that using EPS backfilling for shallow tunnels reduced the vertical stress on the tunnel by up to 85% due to EPS’s lightweight properties and the positive effect of soil arching. The analysis conducted by AbdelSalam et al.^28^ also indicated that the long-term strain in the EPS blocks remains below 1% after 100 years of load application, resulting in an insignificant change in initial elastic modulus. Consequently, long-term maintenance of EPS can be neglected in their application. However, if the long-term strain exceeds 1%, the resulting reduction in the initial elastic modulus must be considered to determine maintenance requirements. Additionally, a study by T.A. Siddik et al.^29^ found that backfilling with EPS geofoam results in more than a 49% reduction in vertical displacement compared to soil backfilling.

In conclusion, their concrete slab and wall thicknesses of 1.0–1.4 m, while this study identifies the concrete thicknesses for the soil backfill alternative ranging from 0.8 to 1.2 m. These findings are consistent with prior results, but the variation arises from the detailed assessment of seismic effects on the soil, which generate additional stress on the tunnel sections, an aspect that lies beyond the scope of this study.

## Life cycle assessment (LCA)

LCA was adopted in this study to assess the environmental impacts of the three alternatives considered, as it provides a structured approach to evaluate resource consumption, energy use, and emissions. This aims at pinpointing areas for improvement and supporting industry and researchers in reducing environmental footprints and decision-making for sustainable development^30^.

The ReCiPe Endpoint (global) 2016 method was chosen due to its comprehensive approach to describing impacts on ecosystems, human health, and resource availability. By breaking down complex environmental indicators into three primary damage categories, it offers an endpoint-oriented perspective that simplifies decision-making. ReCiPe’s global perspective also enables consistent comparison across various processes, making it suitable for assessing environmental impacts in different geographical locations. The environmental impact assessment was carried out using SimaPro (version 9.6.0.1) LCA software due to its extensive capabilities. SimaPro is the most widely used software application for performing LCA and is recognized globally for its flexibility and powerful analytical capabilities. It was integrated with extensive databases such as Ecoinvent that can offer access to high-quality, standardized data, fitting for detailed analysis^31^.

### Goal and scope definition

The goal of this environmental study is to assess and compare the environmental impacts of three road embankment methods: Traditional soil backfilling using dense sand and gravel, EPS geofoam coated with HDPE geomembrane, and EPS geofoam coated with newly developed nanocoating (based on nanocellulose extracted from agricultural waste).

### System boundary

The study covers the life cycle from extraction to end-of-life (cradle to grave), focusing on four main life cycle phases as follows:

- Phase 1: “Extraction of raw materials and manufacturing” including dense sand and gravel, EPS geofoam, HDPE geomembrane, and nanocoating components (nanocellulose, polyvinyl acetate, zinc oxide, and elastic polyurethane).
- Phase 2: “Transportation to the construction site” including the transportation of soil backfilling material, EPS geofoam blocks, geomembrane sheets, and nanocoated EPS geofoam blocks. During the manufacturing process in the factory, EPS geofoam blocks are coated with nanocoating material, allowing them to be transported together in the same transportation medium. In contrast, HDPE geomembrane sheets are transported separately.
- Phase 3: “Construction process of the embankment” including embankment construction techniques for the three alternatives, such as the compaction process for soil backfilling and welding of HDPE geomembrane sheets for waterproofing purposes.
- Phase 4: “End-of-life” includes the landfill and recyclable end-of-life treatment due to its remarkable impact on the environment.

**Fig. 4:**
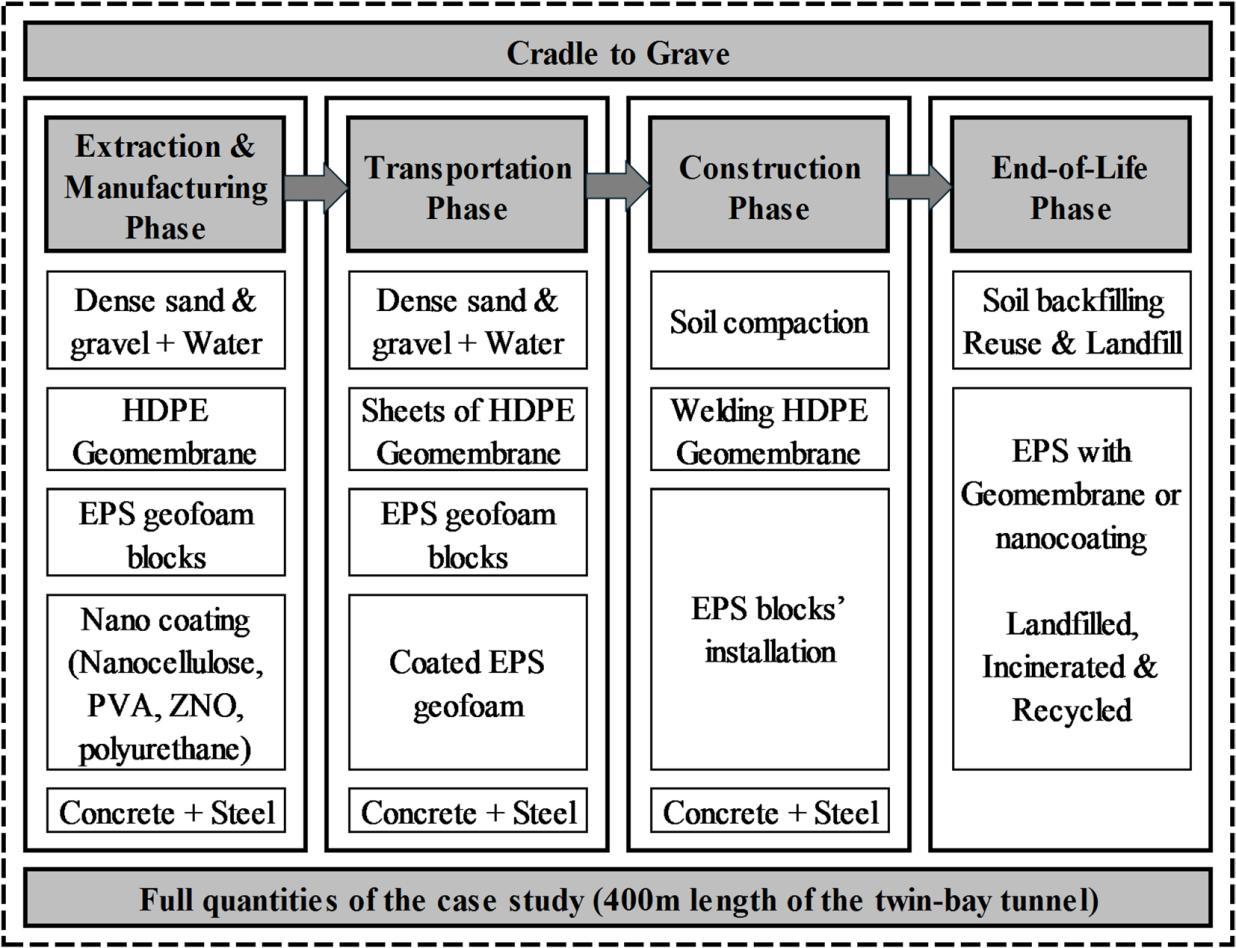
System boundaries.

The environmental impacts during the operation/maintenance phase for the considered case study are similar across all alternatives, making this phase negligible for comparison purposes. LCA is applied to the three alternatives, considering the full quantities of the case study (400 m length of the twin-bay tunnel), to account for material savings in the structure (i.e., concrete and reinforcement steel) that affect the environmental impact assessment. Figures 4 illustrates the system boundaries. The twin-bay tunnel project data was used to define system boundaries and model life cycle processes, including design aspects such as material quantities, construction methods, and logistical requirements for each alternative. The inputs for the coating process of the nanocoated EPS alternative were obtained from experimental laboratory testing. Life cycle phases were simulated using SimaPro software with datasets from Ecoinvent and other sources, covering raw material extraction, manufacturing, transport, and end-of-life treatments. Context-specific assumptions, like realistic transportation distances, were applied to adapt the datasets to the case study.

### Life cycle inventory (LCI)

The life cycle inventory (LCI) in this study evaluates the raw materials, energy flows, and transportation needs for each alternative throughout its life cycle, considering the full material quantities of the tunnel based on the prepared structural design.

#### Raw materials

An inventory analysis was performed for each alternative, and the material quantities needed for the tunnel were calculated and presented in the supplementary Table (A) (Appendix A – Supplementary data). The amounts of concrete and steel reinforcement used in the tunnel structure are considered. The total volume of backfill was 48,800 m^3^. The required quantity of material was calculated by multiplying its density (r) by volume (V) according to Eq. (1):

1 $$\:M=\rho\:\times\:V\:$$

The mass of dense sand and gravel (M_soil_) was calculated based on a bulk density of 20.42 kN/m^3^. The required water for optimal compaction was estimated at an optimum moisture content of 8% of the dry soil density, typical for granular soils like sand and gravel.

2 $$\:{M}_{Soil}={\rho\:}_{b}\times\:V\:=\:20.42\times\:\text{48,800}\times\:100=\text{99,649,600}\:\text{k}\text{g}$$

The mass of EPS geofoam (M_Foam_) was calculated based on a density of 25 kg/m^3^.

3 $$\:{M}_{Foam}=25\times\:\text{48,800}=\text{1,220,000}\:\text{k}\text{g}$$

The mass of HDPE geomembrane (M_Geomembrane_) was calculated based on a density of 930 kg/m^3^ and a thickness of 2 mm. The estimated surface area of 323,544 m^2^ for the HDPE geomembrane included welding/waterproofing edge overlaps.

4 $$\:{M}_{Geomembrane}=930\times\:\left(\text{323,544}\times\:0.002\right)=\text{601,791}\:\text{k}\text{g}$$

For the nanocoating protection layer, the coating mass was calculated based on the density of each component and its percentage in the nanocoating product. The densities of nanocellulose, PVA, and zinc oxide were 120, 1250, and 5,600 kg/m³ respectively, with corresponding percentages of 42%, 50%, and 8%. The thickness of the nanocoating material was 0.7 mm based on^10^. The total calculated quantity of the nanocoating components was 230,252 kg. Furthermore, the EPS block surface was coated with a layer of elastic polyurethane (Kemapoxy 175) with a thickness of 0.1 mm and a density of 1,080 kg/m^3^. The mass of the elastic polyurethane coating was calculated as 31,622 kg and added as a separate layer. The amount of concrete and reinforcement steel was previously determined according to the tunnel embankment design.

It is found that the soil backfilling alternative requires the highest quantities of materials, with about 186,146 tons, given the high mass of dense sand and gravel, along with the needed concrete and steel according to the structural design. The EPS with HDPE geomembrane method has about 32,464 tons of materials. While EPS with the nanocoating technique has the lowest total material amount of about 24,925 tons, it has the potential of being resource efficient compared to all other alternatives.

#### Energy

For the soil backfill alternative, the use of equipment such as a loader is essential for transporting and depositing the dense sand and gravel. The loader bucket capacity was assumed to be 4 m³ per cycle. With a cycle time of 1 min per load, the total number of cycles required to complete the operation was calculated. In addition, the total time required for the loader to complete the soil backfilling was determined based on the cycle time according to Eq. (5). Data input for SimaPro includes the loader’s operational time, the diesel consumption of the machinery, hydraulic oils, and general lubricants needed for the hydraulic systems.

5 $$\:{T}_{1}=\left(\frac{{V}_{b}}{{V}_{c}}\right)\times\:{t}_{sc}$$

Where T_1_ is the equipment total usage time (hr), V_b_: total backfilling volume (m^3^), V_c_ is the single cycle capacity of equipment (m^3^), and t_sc_ is the time consumed per single cycle (hr).

6 $$\:{T}_{1}=\left(\frac{\text{48,800}}{4}\right)\times\:\left(\frac{1}{60}\right)=203.3\:\text{h}\text{r}$$

The environmental impact of the soil compaction operation was assessed by calculating the operating time of diesel equipment for the total backfill volume. A productivity rate of 100 m^3^/hr was assumed according to the commonly used benchmarks^32^. The overall time required for the compaction process was calculated using Eq. (7):

7 $$\:{T}_{2}=\frac{{\text{V}}_{b}}{P}$$

Where T_2_ is the total operation time (hours), V_b_ is the total backfilling volume (m^3^), and P is the productivity rate (m³/hr).

8 $$\:{T}_{2}=\frac{\text{48,800}}{100\:}=488\:\text{h}\text{r}$$

Regarding the second alternative EPS geofoam with geomembrane, EPS is manufactured by the suspension and polymerization of styrene monomers, with additives like flame retardants and plasticizers. Data for EPS production is sourced from the Eco-profiles of the European plastics industry (Plastics Europe), covering over 80% of European production based on the Ecoinvent database (version 3). HDPE geomembranes require significant energy during their installation due to the welding processes. Above the tunnel, it was assumed that a HDPE is applied to each EPS block to ensure full waterproofing, as there were no side walls or existing boundaries to use HDPE for bulk wrapping of the ESP blocks. The energy required for welding the HDPE geomembrane was estimated according to Eq. (9).

9 $$\:{E}_{2w}={P}_{machine}\times\:\left(\frac{n\times\:L}{{S}_{w}}\right)\times\:\:\:{V}_{EPS}$$

Where E_2w_ is the total energy consumption (kWh), P_machine_ is power of the welding machine (kW), “n” is the number of edges per cube, “L” is the length of each edge (m), S_w_ is the welding speed (m/hours), and V_EPS_ is the total volume of EPS blocks (m^3^).

10 $$\:{E}_{2w}\:=\:0.8\times\:\left(\frac{1\times\:7}{48}\right)\times\:\text{48,800}\:=\text{5,693}0.33\:\text{k}\text{W}\text{h}$$

For EPS with nanocoating, the energy consumption for nanocellulose extraction processes was evaluated experimentally based on the power ratings of the devices and their operational times. The primary devices used were an oven, an ultrasonic device, and a magnetic stirrer. The oven consumed 11.27 kWh over 49 h, resulting in the material volume and processing percentage yielding a final energy consumption of 4.34 kWh. The ultrasonicator operated for 30 min with a power rating of 0.1209 kWh, resulting in a total energy of 0.06045 kWh and a final energy of 0.0499 kWh. The magnetic stirrer, running for 6 h with a power rating of 0.6 kWh, had a total energy consumption of 3.6 kWh, which resulted in 1.98 kWh.

Energy inputs were calculated for each process step, whereas the nanocoating application required about 6.37 kWh of energy for nanocellulose extraction processes. It can be noticed that the highest contribution was the energy consumed by the oven due to the time required for drying. Then, the energy consumption for the entire volume was calculated based on the per-unit energy values derived from the experimental data as presented in Eq. (11).

11 $$\:{E}_{3e}={V}_{EPS}\:\times\:\left[\sum\:{r}_{i}\times\:{t}_{i}\times\:\left(\frac{{v}_{d}}{{v}_{n}}\right)\right]$$

Where “E_3e_” is the energy consumption for the nanocellulose extraction (kWh), “V_EPS_” is the volume of coated EPS blocks for the tunnel, “r_i_” is the consumption rate of the device (kWh),” t_i_” is the required time of the extraction process in the device (hours), “v_d_” is the device capacity volume (m^3^), and “v_n_” is the volume of nanocellulose (m^3^).

12 $$\:{\text{E}}_{\text{O}\text{v}\text{e}\text{n}}=0.23\times\:49\times\:\left(\frac{0.042875}{0.016514}\right)=4.34\:\text{k}\text{W}\text{h}$$

13 $$\:{\text{E}}_{\text{U}\text{l}\text{t}\text{r}\text{a}\text{s}\text{o}\text{n}\text{i}\text{c}\text{a}\text{t}\text{o}\text{r}}=0.1209\times\:0.5\times\:\left(\frac{0.02}{0.016514}\right)=0.049914\:\text{k}\text{W}\text{h}$$

14 $$\:{\text{E}}_{\text{M}\text{a}\text{g}\text{n}\text{e}\text{t}\text{i}\text{c}\:\text{S}\text{t}\text{i}\text{r}\text{r}\text{e}\text{r}}=0.6\times\:6\times\:\left(\frac{0.03}{0.016514}\right)=1.981678\:\text{k}\text{W}\text{h}$$

$$\:{\text{E}}_{3\text{e}}=\text{V}\times\:\left({\text{E}}_{\text{O}\text{v}\text{e}\text{n}}+{\text{E}}_{\text{U}\text{l}\text{t}\text{r}\text{a}\:\text{S}\text{o}\text{n}\text{i}\text{c}}+{\text{E}}_{\text{M}\text{a}\text{g}\text{n}\text{e}\text{t}\text{i}\text{c}\:\text{S}\text{t}\text{i}\text{r}\text{r}\text{e}\text{r}}\right)=\:\text{48,800}\times\:6.372409=\text{310,974}\:\text{k}\text{W}\text{h}$$

Regarding the manufacturing processes, a homogenizer with a rating of 7.5 kW was used based on industry-standard specifications. The mixing process operated for 1 h per batch, which results in a total energy consumption of 7.5 kWh per batch. The homogenizer had a capacity of 2,000 L per batch. The required energy to mix this total volume was calculated to be 863,445 kWh according to Eq. (15).

15 $$\:{E}_{3\,m}=\left(\frac{P\times\:t}{v}\right)\times\:{V}_{n}$$

Where “E_3m_” is the energy consumption for the mixing process (kWh), “P” is the power of the fluid homogenizer (kW), “t” is the time of mixing (hours), “v” is the capacity volume (liters), and “V_n_” is the volume of nanocoating (liters). The volume of nanocoating was estimated based on its thickness of 0.7 mm, knowing that the surface area of a unit EPS block is equal to 6 m^2^. The energy consumed for the mixing process was calculated as follows:

16 $$\:{E}_{3\,m}=\left(\frac{7.5\times\:1}{2000}\right)\times\:\left(0.0007\times\:6\times\:\text{48,800}\times\:1000\right)=768.6\:\text{k}\text{w}\text{h}$$

The automotive painting process was adopted to model the nanocoating painting activity for EPS geofoam blocks. The surface area of the EPS geofoam required for painting was calculated to be 292,800 m² based on its total volume. In addition, the Ecoinvent database was used to get energy for other materials, including polyvinyl acetate (PVA), zinc oxide (ZnO), elastic polyurethane, and geomembrane extraction and manufacturing.

#### Transportation

To assess the environmental impact of transportation in the LCA method, the ton-kilometer (ton-km) method was used to determine the transportation burden by multiplying the mass of the transported material by the distance traveled according to Eq. (17)^33^.

17 $$\:{\text{T}}_{\text{i}\text{m}\text{p}\text{a}\text{c}\text{t}}=\text{M}\times\:\text{D}$$

Where T_impact_ is the transportation impact (ton-km), M is the mass (ton), and D is the distance (km). The load factor (LF) concept is crucial for efficient transportation. To reflect real-world logistical characteristics, load factors were applied in SimaPro based on each material’s density. The used LF values were 100% for dense sand and gravel, 80% for HDPE geomembrane, and 20% for EPS geofoam, aligning with their physical characteristics and typical loading capacities. A uniform transportation distance of 50 km was assumed for all three alternatives, with an empty return scenario^34,35^. Transport was modelled according to the “Transport, truck” dataset for Ecoinvent v3.8, where there is an average global process and a functional unit of 1 tonne kilometre (tkm). This dataset is assumed from fuel usage from primary data for small vehicles (< 10t), medium-sized (10–20t), and large-sized (> 20t) trucks. Calculated emissions are weighted averages of road types relating to typical patterns of truck usage. European emission regulations are used to account for the varying amounts of regulated pollutants such as NOx, CO, and particulate matter.

It was found that the total transportation impact of soil backfilling, EPS with HDPE geomembrane, and EPS with nanocoating is 8,908,721, 1,623,197, and 1,246,249 ton-km, respectively. The calculations highlight a significant reduction in transportation demands for EPS with nanocoating by 86% and 23% compared to soil backfill and to EPS with HDPE geomembrane, mainly due to its lower mass. Additionally, applying the nanocoating directly to the EPS geofoam at the manufacturing facility eliminated the need for separate transportation steps, optimizing truck volume utilization. Supplementary Tables (B) and (C) present the transportation impacts of the alternatives and all quantities, processes, and databases used in SimaPro (Appendix A – Supplementary data).

#### End-of-life

The end-of-life (EoL) stage involves material treatment, with disposal scenarios designed to represent common practices for each assembly as described in literature on geosynthetics, polymer recycling, and waste management. These processes are represented as global market activities using Ecoinvent v3.8 datasets, reflecting the average worldwide mix of residual waste treatment methods. Each activity begins at the gate of the respective process burden source and ends where the service delivery occurs. The processes are represented to include relevant emissions and resource consumption associated with landfilling and incineration.

The reuse of soil backfill is constrained by factors such as contamination, logistical challenges, and other obstacles, resulting in significant dependence on landfill disposal. These challenges encompass regulatory, organizational, logistical, and material quality concerns^36^. Consequently, it was estimated that 60% of the soil backfill would be disposed of in landfills, while the remaining 40% would be repurposed for backfilling or leveling applications.

Several studies presented innovative approaches to enhancing the reuse and recycling of EPS blocks in construction. Leão et al. demonstrated that recycled EPS could substitute up to 30% of sand in rendering mortars, improving both fluidity and mechanical properties^37^. Mumbach et al. achieved over 92% recovery of EPS using dissolution and polymerization, maintaining its desirable properties^38^. Bartlett et al. highlighted the reuse potential of EPS geofoam in infrastructure projects, such as road embankments and retaining walls^39^. Achilias et al. investigated the chemical recycling of polystyrene, breaking it down into styrene for reuse^40^. Furthermore, it was reported that 11% of global waste is incinerated for energy recovery^41^. Consequently, in this study, it was assumed that 30% of the material would be landfilled, 10% to incineration, and 60% would be reused for the case of EPS geofoam with HDPE geomembrane or nanocoating. After serving its lifecycle as an embankment material, EPS geofoam can be ground, cleaned, and processed for reuse in infrastructure projects. This includes partially substituting traditional construction materials like sand in a mortar mixture or using it as a lightweight fill-in for embankments.

EPS geofoam faces end-of-life challenges due to its low density, high transportation costs, and contamination from additives, which hinder recycling. Despite efforts to establish recycling plants, economic and logistical issues lead to large quantities ending up in landfills. Coated EPS adds complexity to recycling and disposal. However, bio-based nanocoatings offer a potential solution by increasing biodegradability and reducing environmental impact, with advancements suggesting viable alternatives in the future^42^.

### Life cycle impact assessment (LCIA)

The ReCiPe 2016 Endpoint method was employed to evaluate the environmental impacts by translating the LCI data into three main categories: human health, ecosystems, and resource availability. ReCiPe 2016 was selected for its comprehensive approach to LCIA, integrating both midpoint and endpoint indicators to provide a balanced evaluation of environmental impacts. In contrast to other methods, ReCiPe 2016 places emphasis on endpoint indicators, such as DALY (Disability-Adjusted Life Years), species-year, and USD (economic valuation), making it relevant for studies focused on human health, biodiversity conservation, and economic sustainability, and enabling effective communication of results. The endpoint orientation of ReCiPe 2016 aligns well with the study’s sustainability objectives by translating impacts into measurable outcomes. This alignment ensures the study meaningfully contributes to broader sustainable development goals^31,43^. The analyzed impact parameters with descriptions and abbreviations are presented in Supplementary Table (D) in Appendix A (Supplementary data).

### Environmental results and discussion

**Fig. 5:**
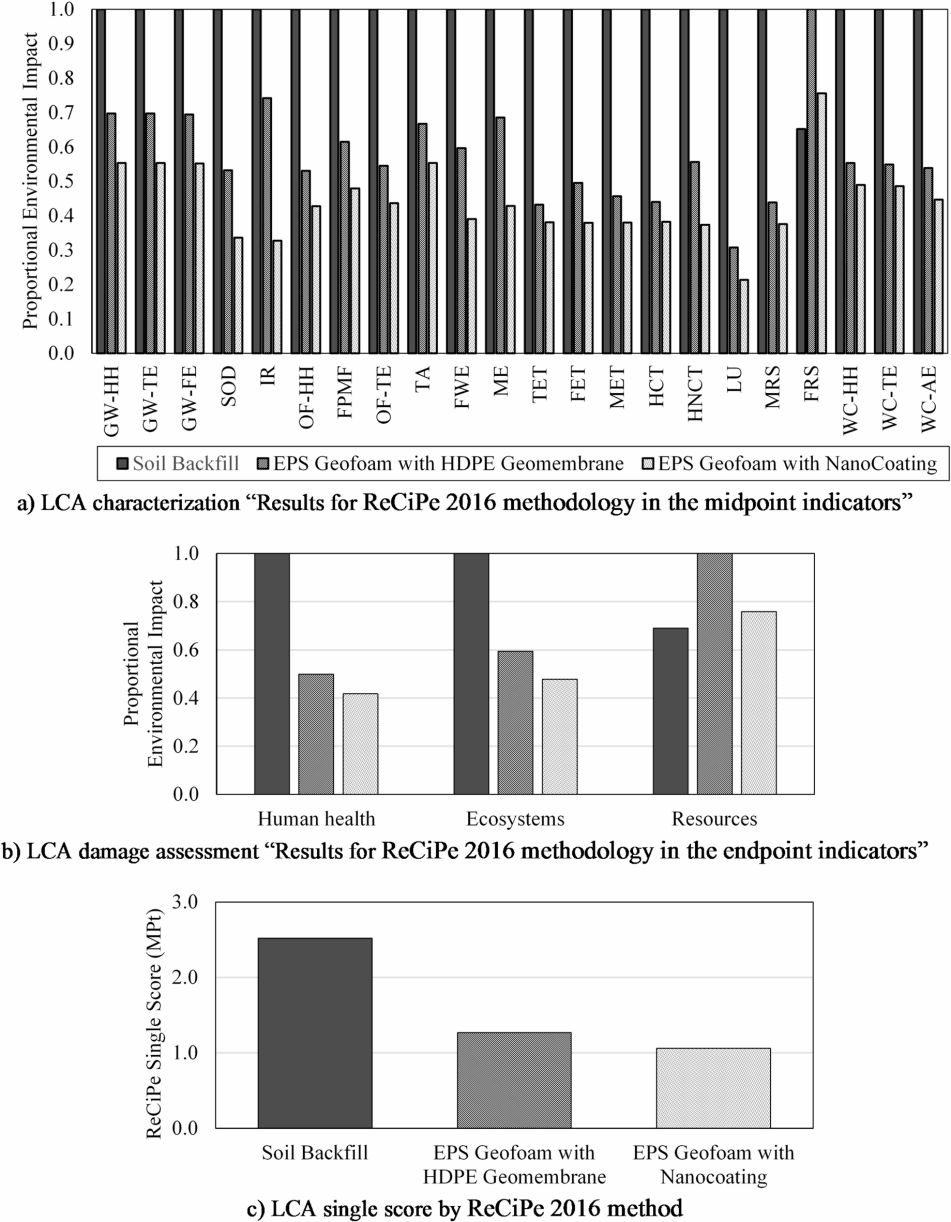
Results of the environmental impacts using ReCiPe 2016 method.

Results of LCA were analyzed across three damage categories: Human Health, Ecosystems, and Resources. Characterization results in Figure 5a reveal significant disparities in environmental impacts across the three embankment alternatives. EPS geofoam with nanocoating demonstrates the lowest impact, followed by EPS with HDPE geomembrane, and this is for all impact parameters except for the “fossil resource scarcity” parameter (FRS), where soil backfilling has the lowest impact. For global warming potential (GWP), the nanocoated EPS recorded the lowest impact on human health at 11.38 DALY compared to a significantly higher value for soil backfill at 20.57 DALY. Similarly, fine particulate matter formation (FPMF), a key contributor to human health damage, was reduced from 17.19 DALY in soil backfill to 8.24 DALY in the nanocoated EPS alternative, emphasizing its potential to mitigate air pollution-related health risks. In terms of toxicity, human carcinogenic toxicity (HCT) for the nanocoated EPS was 62% lower than that of soil backfill due to reduced emissions during material processing. For resource scarcity, the nanocoated EPS alternative exhibited the lowest impact on mineral resource scarcity (MRS) but was slightly higher in fossil resource use compared with soil backfill due to its polymeric nature. Ecosystem-related impacts, such as terrestrial acidification (TA) and freshwater eutrophication (FWE), followed a similar trend with nanocoated EPS consistently outperforming soil backfill. Results highlight the vital role of lightweight materials in reducing environmental impacts and maintaining functional performance.

Figure 5b demonstrates that the human health category for soil backfill recorded the highest damage at 148.58 DALY (disability-adjusted life years), more than twice that of EPS with nanocoating at 62.00 DALY due to the higher emissions of fine particulate matter and carcinogenic substances during the life cycle of soil backfill. Similarly, for the ecosystems category, soil backfill contributed with 125.35 × 10^− 3^ species.yr compared to 59.95 × 10^− 3^ species.yr for EPS with nanocoating, highlighting the ecosystem benefits of using lightweight engineered materials. For resources, EPS geofoam with HDPE geomembrane showed slightly higher impact than EPS with nanocoating due to the additional material requirements of the HDPE layer.

Normalization further emphasizes the environmental advantages of EPS with nanocoating, particularly in terms of human health and ecosystem impacts. For the human health category, the nanocoating assembly recorded the lowest normalized value of 2585.43, a reduction of 16% compared to EPS with HDPE geomembrane of 3091.60, and 58% lower than soil backfill of 6195.58. These reductions highlight the minimized health-related burdens associated with emissions from nanocoating assembly, streamlined manufacturing, and transportation processes. In the ecosystems category, the nanocoating assembly again demonstrated the lowest impact of 40.53, reflecting a 20% reduction compared to the geomembrane assembly of 50.36, and a 52% reduction relative to the soil backfill of 84.74. For resources, the geomembrane assembly exhibited the highest impact of 64.67, likely due to the additional material and energy required for the HDPE layer.

Finally, the single score gives a clear indication of the overall environmental performance of each alternative. This holistic metric considers the cumulative effects of emissions, resource depletion, and ecological disruption and gives an insight into the wider consequences of each backfill approach. The LCA single score in Figure 5c reveals that EPS with nanocoating exhibited the lowest overall environmental impact of 1.06 MPt, followed by EPS with HDPE geomembrane of 1.27 MPt, and finally soil backfill of 2.52 MPt. This trend highlights the benefits of incorporating nanocoating into EPS blocks, which reduces impacts across all damage categories. It was evident that reducing the amount of reinforced concrete by using EPS geofoam blocks with nanocoating or HDPE geomembrane significantly lowers the environmental impact score for the EPS geofoam alternative.

## Life cycle costing (LCC)

According to ISO 15686-5:2017^44^, LCC includes all relevant costs to guide decision-making processes toward economically feasible solutions. By covering the whole financial implications, this approach ensures a comprehensive assessment that aligns with long-term infrastructure planning and sustainability goals^18^. Accordingly, LCC was employed to assess the economic feasibility of the three backfilling alternatives considered in this case study. In addition, the associated health-related costs are estimated using the value of statistical life (VSL) method based on the DALYs obtained from the LCA results^45^. The analysis included materials, transportation, construction, end-of-life, and health costs.

### Cost breakdown

A cost breakdown for material extraction and manufacturing, transportation, construction, and end-of-life phases associated with the three alternatives is conducted and summarized in Table (2). A transportation distance of 50 km was assumed for all alternatives. The necessary data was gathered from various sources, such as the structural design of the case study, material specifications, transportation rates, current local market prices (US dollars $), LCA analysis, and end-of-life costs. Supplementary Table (E) presents the detailed cost breakdown for the three alternatives (Appendix A – Supplementary data). The total cost of each component (C_t_) is calculated according to Eq. (18).

18 $$\:{\text{C}}_{\text{t}}=\sum\:_{i=1}^{n}{u}_{i}\times\:{Q}_{i}$$

Where “u_i_” is the unit cost for the item ($), “Q_i_” is the quantity of the item, and “n” is the number of items for each component/alternative.

### Value of statistical life

The analysis integrated the VSL method to evaluate the health-related costs associated with each alternative, alongside their direct LCC. Using DALYs obtained from LCA, monetary value of health impacts was calculated^46^. The health-related cost was calculated using Eq. (19):

19 $$\:{\text{C}}_{\text{h}\text{e}\text{l}\text{a}\text{t}\text{h}}\:={\text{D}}_{\text{D}\text{A}\text{L}\text{Y}\text{S}}\times\:{\text{C}}_{\text{V}\text{S}\text{L}}$$

Where C_health_ is the health-related cost ($), D_DALYS_ is the DALYs, and C_VSL_ is the VSL cost of $37,500. These costs were added to the traditional LCC to obtain the adjusted Life Cycle Cost (ALCC). Table 2 summarizes the DALYs and health-related costs for the three alternatives, providing insights into their comparative performance. The results illustrate that EPS with nanocoating achieves the lowest health-related cost and ALCC among the other alternatives. This is due to its lower DALYs, resulting from reduced environmental impacts across its life cycle stages. By contrast, soil backfill exhibits the highest ALCC.

**Table 2:** Cost analysis for the three alternatives.

Parameter	Soil backfill	EPS geofoam with HDPE geomembrane	EPS geofoam with nanocoating
Materials Extraction and Manufacturing Cost ($)	5,542,560	7,214,849	6,210,644
Transportation Cost ($)	498,248	120,129	117,120
Construction cost ($)	233,425	47,558	37,195
End-of-life cost ($)	179,369	37,711	30,675
**Life cycle cost “LCC” ($)**	**6**,**453**,**602**	**7**,**420**,**247**	**6**,**395**,**634**
Human Health (DALY)	148.58	74.14	62.00
Health Related Cost ($)	5,571,566	2,780,215	2,325,029
**Adjusted LCC ($)**	**12**,**025**,**168**	**10**,**200**,**462**	**8**,**720**,**663**

### LCC results and discussion

The high density of dense sand and gravel significantly affected material quantities for soil backfilling, leading to increased transportation costs due to the large amounts required. Additionally, the construction phase added considerably to the total cost, while soil compaction required extra time and equipment. Moreover, the increased weight of dense sand and gravel necessitated higher amounts of reinforced concrete for the tunnel’s structural elements.

Using EPS with HDPE geomembrane offered advantages due to EPS’s low weight, but incurs higher initial material costs because of the high unit price of EPS geofoam and the expensive HDPE sheets. Installation costs, including placement, welding, and energy consumption, also contributed to the overall construction expenses. The placement and welding of the HDPE geomembrane sheets, including welding energy, laborers, and machines, reflect the energy-intensive nature of this process. Additionally, transportation for both EPS blocks and HDPE sheets was less costly compared to the transportation of dense sand and gravel, due to the lighter weight of EPS. Overall, while the EPS with geomembrane alternative offered cost-effective transportation and construction due to its lightweight, the initial material cost and energy-intensive installation process contributed to a significant portion of its total LCC.

The EPS with nanocoating is an innovative coating solution that addresses the limitations of traditional methods while introducing new cost factors. The initial material costs were significantly affected by the high prices of PVA, zinc oxide, and epoxy, but transportation costs were reduced due to the lightweight nature of the coated EPS. The manufacturing phase of nanocoating added expenses, including energy consumption for mixing and the labor-intensive painting process. However, construction costs were lowered due to reduced concrete and steel reinforcement requirements. Despite the high initial investment, this alternative offered lifecycle benefits, making it a sustainable choice for infrastructure projects.

**Fig. 6:**
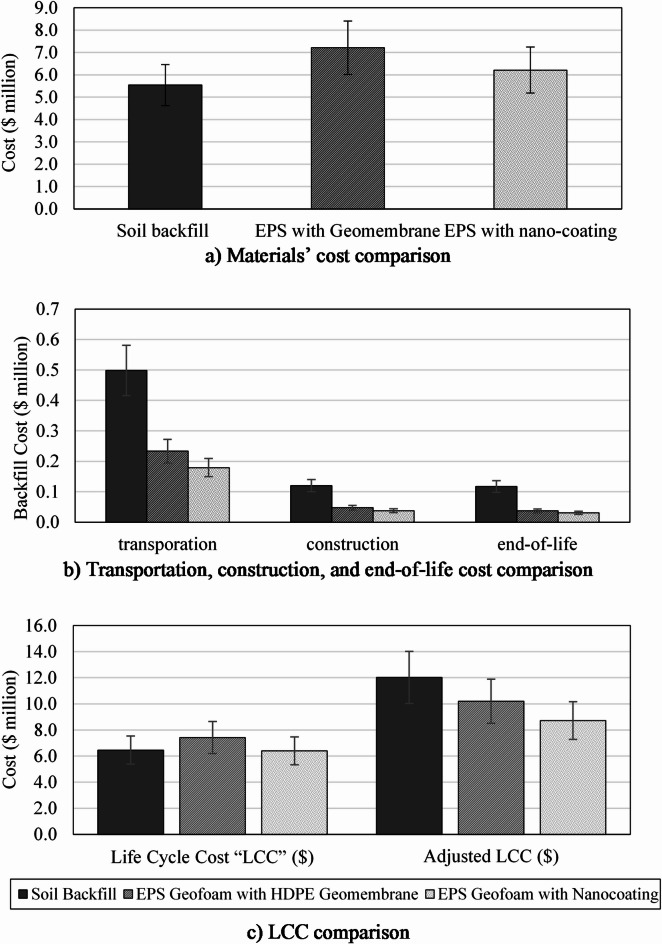
Cost Comparison.

The results highlight significant cost differences driven by material properties, transportation requirements, construction processes, and end-of-life management. Both EPS geofoam with HDPE geomembrane and nanocoating had total LCC less than soil backfilling by about 15.17% and 27.72%, respectively. Furthermore, the EPS with nanocoating had a total LCC less than the EPS with HDPE geomembrane by about 14.79%. Soil backfilling had the lowest initial material cost due to the relatively low cost of sand, as shown in Figure 6a. From the figure, the EPS geofoam blocks with HDPE geomembrane incurred the highest material cost.

Soil backfilling incurred the highest construction cost due to the complicated soil compaction process and the increased need for concrete and steel reinforcement for the tunnel. In contrast, the construction cost for EPS with geomembrane was driven by the labor and energy required for geomembrane installation. EPS with nanocoating had the lowest construction costs due to reduced concrete and steel reinforcement, in addition to a simpler coating process. Soil backfilling also had the highest end-of-life cost because of disposal requirements. Figures 6b illustrates a comparison between the cost of transportation, construction, and end-of-life for the three alternatives.

EPS with nanocoating demonstrated the lowest total LCC despite its high initial material and manufacturing costs, as presented in Figure 6c. Its lightweight properties reduced transportation and construction expenses. The differences in LCC among the alternatives were driven by material density, availability, and lifecycle characteristics. Soil backfilling was cost-effective initially but requires more transportation and structural reinforcement due to its heavy weight. EPS with geomembrane was constrained by the high initial cost and limited availability of HDPE, in addition to its complex end-of-life processes. EPS with nanocoating provided a balance with reduced transportation and construction costs.

It is important to recognize that economic feasibility varies significantly across regions, influenced by factors such as resource availability, labor expenses, and environmental regulations. Areas rich in local materials and skilled labor tend to have reduced costs, while regions reliant on imports or workforce training may experience higher expenditures. Labor costs also differ, with developing regions often providing more affordable labor but potentially requiring skill enhancement^47^. Stricter environmental regulations can lead to higher compliance costs, whereas lenient policies might result in future challenges. Additionally, well-established infrastructure minimizes logistical expenses, and local economic conditions impact purchasing power and market potential. Each case demands a tailored evaluation, given the complexity of these interconnected elements.

## Sensitivity analysis

Sensitivity analysis is a critical tool in assessing the robustness and reliability of LCA and LCC outcomes, particularly in studies involving significant assumptions or uncertain input parameters. It involves systematically varying key inputs to evaluate their impact on the results, ensuring that uncertainties or subjective choices do not unduly influence findings.

In this study, a sensitivity analysis was performed to assess how key parameters affect road embankment alternatives’ environmental and economic performance using a one-at-a-time (OAT) approach, where one variable is changed while keeping others constant, for simplicity and interpretability^48^. Concrete and reinforcement steel, used in the tunnel’s construction, were identified as major contributors to both environmental impact and cost. Thus, a model excluding the tunnel’s structural design was applied. Secondly, various transportation distance scenarios and end-of-life scenarios were tested to determine their effect on the environmental impacts and costs. Finally, other factors influencing results, such as climate conditions and traffic loads, are discussed. These analyses aimed to gain a deeper understanding of the environmental and economic impacts associated with material selection and transportation logistics for embankment applications.

### The influence of the tunnel’s structural design

**Fig. 7:**
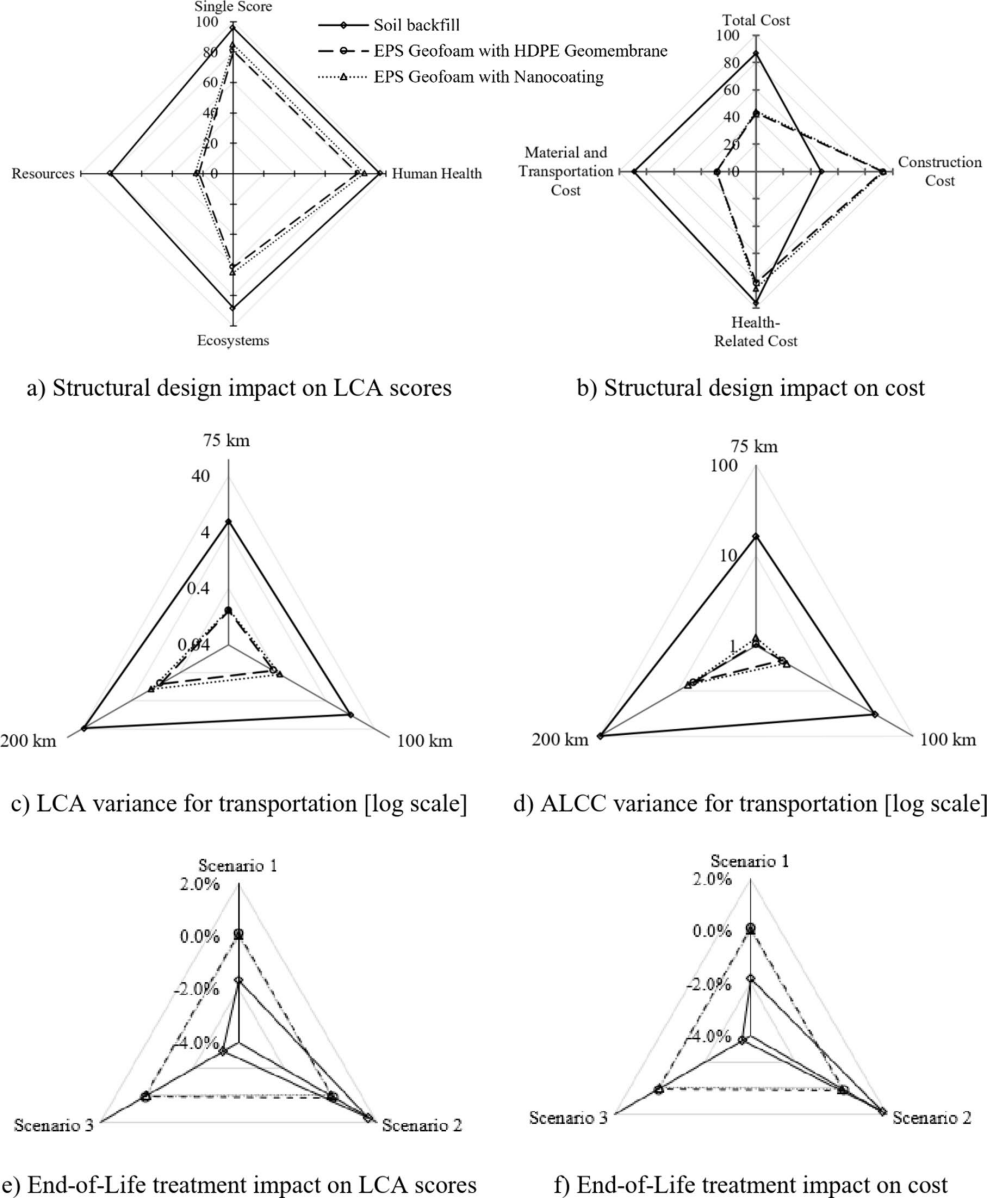
Variances due to the impacts of structural design, transportation distance, and end-of-life treatment.

The analysis assesses three methods, with and without concrete and steel, across human health, ecosystems, and resource depletion. Figures 7a shows relative changes in environmental scores, including the single score, human health score, ecosystems score, and resource depletion score. Scores significantly decreased for soil backfill by 96%, 96%, 88%, and 81%, respectively. For EPS with HDPE geomembrane, the scores dropped by about 81%, 82%, 62%, and 23%, respectively. For EPS with nanocoating, the scores decreased by around 85%, 86%, 65%, and 25%, respectively. The most significant drop was seen in the soil backfill method and the single scores. The smallest drop was observed in the resource category.

The cost analysis evaluates the alternatives, with and without concrete and steel. Figures 7b illustrates the relative changes in the cost, including the cost of materials and transportation, construction, health-related, and total cost. For soil backfill, the costs decreased by approximately 85%, 48%, 96%, and 87%, respectively. For EPS geofoam with HDPE geomembrane, the costs dropped by about 29%, 93%, 82%, and 43%, respectively. For EPS geofoam with nanocoating, the costs decreased by around 29%, 93%, 86%, and 44%, respectively. The most significant drop was seen in the soil backfill method, particularly in the health-related cost, which matches the results of the sensitivity analysis of LCA in the previous section. The smallest drop was observed in the materials cost of EPS geofoam with HDPE geomembrane or nanocoating.

LCA and LCC trends indicated that excluding concrete and steel reinforcement significantly altered the environmental impact ranking and cost, shifting soil backfill from the highest to the lowest impact and cost option. However, this introduces a methodological limitation, as the choice of embankment material directly affects the structural design and concrete quantities. Therefore, it is crucial to include these materials in the comparative analyses for a comprehensive and accurate evaluation. Excluding concrete and steel was not feasible, as the type of backfill material directly influenced the design and structural requirements of the twin-bay tunnels, affecting their load-bearing capacity, settlement behavior, and overall stability.

### The influence of the transportation distance

The analysis aimed to evaluate the impact of backfilling material transportation on overall environmental and economic performance. Several scenarios with incremental transportation distances of 25 km, 50 km (baseline), 75 km, 100 km, and 200 km were investigated, excluding concrete and steel reinforcement materials. Figures 7c shows the variation in the LCA single score for each alternative across these transportation scenarios. It was found that the environmental impact of using the soil backfill alternative significantly increased with distance compared to the other EPS alternatives. EPS-based alternatives became more competitive for longer distances due to the minimal increase in environmental impact with the increasing transportation distance.

From Figure 7d, the soil backfill alternative exhibited a rise in total adjusted costs ALCC as transportation distance increased due to its high density and weight, significantly contributing to fuel consumption and related health impacts. The ALCC relative difference for the soil backfills increased sharply with transportation distance, reaching a 98.14% rise at 200 km, emphasizing the high-cost impact of long transport distances for conventional soil. This is accompanied by a 37.33% increase in the single score and a 36.50% rise in DALYs, indicating greater environmental and public health burdens. In contrast, the EPS alternatives, both with HDPE geomembrane and nanocoating, displayed a more gradual, low, and linear cost increase, reflecting their lightweight properties and lower transportation cost sensitivity. For the EPS with HDPE geomembrane, the ALCC increase at 200 km was 6.29%, with DALYs and the single score each rising by 0.98%.

Although the environmental and health impacts were less severe for EPS compared with the soil backfill, longer transportation distances still imposed noticeable burdens. The EPS with nanocoating showed the least impact across all alternatives, with a 7.35% rise in ALCC, a 1.54% increase in DALYs, and a 1.53% increase in the single score at 200 km, suggesting that the nanocoating option maintains superior environmental and public health performance even as transportation distances increase.

### The influence of the End-of-Life treatment

The analysis aims to evaluate the environmental and cost impacts of various backfilling materials at their End-of-Life (EoL). Four EoL treatment scenarios were considered, excluding concrete and steel reinforcement materials, based on waste treatment practice and literature. The base scenario involved 40% reuse and 60% landfill for soil backfill, and 60% recycling, 30% landfill, and 10% incineration for EPS geofoam alternatives. The first scenario included 50% reuse and 50% landfill for soil backfill, and 20% recycling, 50% landfill, and 30% incineration for EPS geofoam alternatives. The second scenario comprised 30% reuse and 70% landfill for soil backfill, and 10% recycling, 70% landfill, and 20% incineration for EPS geofoam alternatives. The third scenario comprised 60% reuse and 40% landfill for soil backfill, and 10% recycling, 80% landfill, and 10% incineration for EPS geofoam alternatives. Based on Fig. 7e and f, changes in EoL treatment had no significant influence on environmental or economic impacts. For soil backfilling, the third scenario demonstrated the highest variation, with improvements of 3.3% (LCA single score) and 3.62% (ALCC) compared to the base scenario, due to the high reuse percentage. Conversely, the second scenario for soil backfill showed declines of 1.65% (LCA single score) and 1.81% (ALCC), attributed to its high landfill percentage. Among the two EPS alternatives, EPS with HDPE geomembrane exhibited the greatest variation in the first scenario, with increases of 0.12% and 0.08% for LCA single score and ALCC, respectively, due to a high incineration percentage. Nanocoated EPS, on the other hand, showed the least variation across all scenarios.

### The influence of soil properties, climate conditions, traffic loads, and lifespan

Differences in soil properties, climate conditions, and traffic loads play a critical role in determining whether to use natural soil or EPS blocks for embankments. Natural soil, when placed on weak or compressible ground with low bearing capacity, may experience significant settlement, making the lightweight EPS blocks a better choice to reduce settlement and maintain stability. In areas with high groundwater levels or frequent heavy rainfall, natural soil embankments are more susceptible to erosion or waterlogging, while EPS blocks, being resistant to water absorption, are less affected by such conditions. Additionally, under heavy traffic or dynamic loads, natural soil may require substantial reinforcement to prevent deformation, whereas the reduced weight of EPS blocks allows for better load distribution. Therefore, selecting the appropriate embankment material requires careful evaluation of these factors to ensure stability and long-term performance.

Throughout their lifespan, natural soil and EPS geofoam blocks demonstrate distinct characteristics due to their inherent properties. Natural soil embankments are prone to settlement, particularly on weak ground or when subjected to heavy loads, requiring regular maintenance. Their durability can diminish over time due to factors such as water infiltration, making effective drainage systems essential to preserve stability. Conversely, EPS geofoam blocks offer excellent durability with minimal maintenance, as they are resistant to water absorption and environmental wear. Their lightweight composition greatly reduces settlement risks and ensures reliable performance under various load conditions, making them an ideal choice for demanding environments.

### Comparison to previous studies

When comparing the findings of this research to previous studies, it was observed that most of the results align, considering the system boundaries, assumptions, and research limitations. For instance, a study conducted in Japan investigated lightweight composite geomaterials, including EPS, by assessing their environmental and economic impacts, considering the external costs related to greenhouse gas (GHG) emissions and pollutants. It showed potential for reducing environmental burdens and enhancing ecosystem services compared to traditional methods^14^. Another study examined the life cycle of EPS in Malaysia, highlighting global warming potential as the most significant environmental impact, with manufacturing being the primary contributing phase^15^. In addition, Samuelsson et al. concluded that material transport and soil excavation were major contributors to the climate impact^20^.

From an economic perspective, Ito et al. revealed that EPS blocks had a high total LCC, primarily driven by the cost of raw materials, because over 90% of the EPS manufacturing cost was attributed to raw material collection, but utilizing recycled embankment materials proved effective in lowering this expense. In contrast, the conventional cut-and-fill method exhibited relatively low LCC^19^. Similarly, Dormohamadi et al. identified earthen systems as the most cost-effective option, largely due to the use of local materials and minimal processing requirements. However, transportation emerged as a significant cost factor^17^.

### Nanocoated EPS for other infrastructure applications

Nanocoated EPS geofoam is a viable alternative for various infrastructure applications, including bridge abutments, retaining walls, and railway embankments. Its lightweight nature reduces soil pressure and structural requirements, leading to significant savings in material quantities, labor, and construction time. Environmentally, the nanocoating, derived from agricultural waste, provides sustainable protection for EPS geofoam with a lower environmental impact compared to conventional methods. This makes it a sustainable solution for these infrastructure applications. In bridge abutments, using EPS minimizes settlement and lateral loads, enhancing structural stability. For retaining walls, its high compressive strength and durability reduce the need for extensive reinforcement. In railway embankments, it mitigates ground deformation and improves load distribution.

## Conclusions and recommendations

Engineers are increasingly confronted with the challenge of designing and constructing sustainable infrastructure that minimizes environmental impacts while remaining cost-effective. In light of this, the development of roadway embankments requires a strong focus on sustainability, which includes not only mitigating environmental effects but also ensuring economic viability.

This research seeks to analyze and compare the environmental effects and economic feasibility of three different road embankment techniques. The first technique involves traditional backfilling using dense sand and gravel, a widely used and conventional method. The second method employs modern expanded polystyrene (EPS) geofoam blocks, covered with a high-density polyethylene (HDPE) geomembrane. This approach is recognized for its lightweight properties and potential to reduce pressure on underlying structures. The third and most innovative option utilizes EPS geofoam blocks that is coated with a specially developed nanocoating material, which aims to enhance durability and sustainability while minimizing environmental impact. To evaluate these techniques, Life Cycle Assessment (LCA) and Life Cycle Costing (LCC) methods were employed.

These approaches allow for a thorough analysis of the potential environmental impacts and economic feasibility associated with a specific real-world project, a twin-bay tunnel. The Life Cycle Assessment considers the entire life span of the project, from the extraction of raw materials and production processes to construction, use, and eventual disposal or recycling. Advanced tools were utilized in the analysis, including SimaPro software for modeling and interpreting life cycle data, the ReCiPe Endpoint method which provides a comprehensive assessment of environmental impacts, and the value of statistical life (VSL) method for calculating health-related costs linked to environmental degradation.

The findings from this research reveal that the EPS geofoam with nanocoating technique has the most favorable outcomes in terms of both environmental sustainability and cost-effectiveness. Specifically, this method achieves an LCA single score of 1.06 MPt, indicating minimal environmental impact, while also realizing cost savings of approximately 14.5% and 27.5% when compared to EPS geofoam with HDPE geomembrane and traditional soil backfilling methods, respectively. In contrast, traditional soil backfilling results in the highest environmental impact and cost, reflected in a single score of 2.52 MPt. The EPS geofoam covered by HDPE geomembrane ranks intermediate with a score of 1.26 MPt, although it does demonstrate a 15.17% cost reduction relative to the soil backfilling technique. In addition to the primary analysis, a sensitivity analysis was conducted to identify and evaluate the most significant variables influencing the outcomes. This analysis examines how different scenarios or assumptions can impact the results, thereby providing further insight into the robustness and reliability of the findings. From the sensitivity analysis, it was found that excluding changes in the design and its effects on the amounts of reinforced concrete used has shifted the soil backfill alternative from the highest to the lowest environmental impact, highlighting the need to include structural design considerations for accurate comparison between embankment alternatives. Furthermore, the EPS geofoam with nanocoating option continues to offer superior environmental and public health performance, even when transportation distances increase.

Overall, this research offers a detailed framework for assessing sustainable infrastructure options in civil engineering, equipping decision-makers with essential information to make informed choices that align with sustainability goals and economic considerations. In light of the limitations identified in this research, it is recommended that similar studies be undertaken across a range of different infrastructure applications to enhance the understanding of the relevant issues. Additionally, future research should consider incorporating the operational phase of infrastructure, as this aspect may have significant implications based on the type of infrastructure examined. An in-depth analysis of both the construction and operational phases could yield valuable insights, contributing to improved infrastructure performance and sustainability.

## Electronic supplementary material

Below is the link to the electronic supplementary material.

Supplementary Material 1

## Data Availability

All data generated or analyzed during this study are included in this published article [and its supplementary information files].

## Abbreviations

ALCC: Adjusted life cycle cost
CO_2_: Carbone dioxide
DALY: Disability-adjusted life years
EoL: End-of-life
EPS: Expanded polystyrene
GHG: Greenhouse gas
HCl: Hydrochloric acid
HDPE: High-density polyethylene
LCA: Life cycle assessment
LCC: Life cycle costing
LCI: Life cycle inventory
LCIA: Life cycle impact assessment
NaOH: Sodium hydroxide
PVA: Polyvinyl acetate
SCB: Sugarcane bagasse
SDGs: Sustainable development goals
VSL: Value of statistical life
ZnO: Zinc oxide
