# Supplementary material for: A comparative analysis of nanocoated expanded polystyrene for sustainable infrastructure applications
Source: Sci Rep. 2025 May 17;15:17168. doi: 10.1038/s41598-025-01257-y (PMC12085576; doi:10.1038/s41598-025-01257-y)
Supplement: Supplementary file 1 — Supplementary Material 1 [file 41598_2025_1257_MOESM1_ESM.docx]

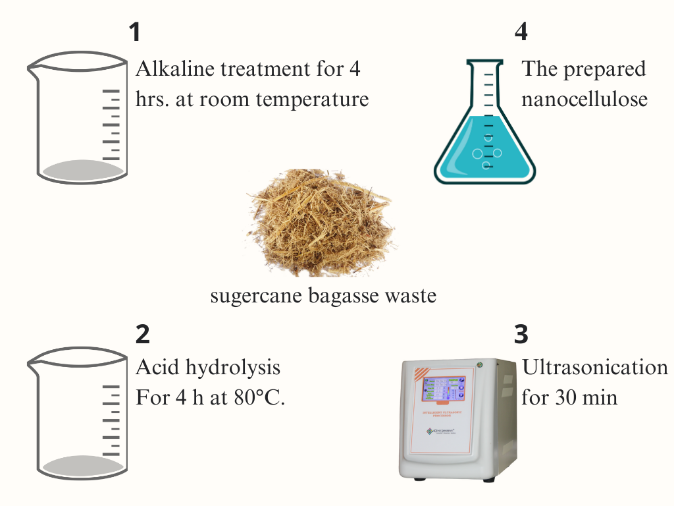


Supplementary Figure A. Nanocellulose extraction processes

Supplementary Table (A): Amounts of raw materials for LCI

| **Raw materials** | **Materials’ Quantities (kg)** | | |
| --- | --- | --- | --- |
|  | **Soil backfilling** | **EPS geofoam with Geomembrane** | **EPS geofoam with Nanocoating** |
| Dense Sand & Gravel | 99,649,600 | - | - |
| Water | 7,971,968 | - | - |
| EPS Geofoam | - | 1,220,000 | 1,220,000 |
| HDPE geomembrane sheets | - | 601,791 | - |
| Nanocoating (Nanocellulose) | - | - | 96,706 |
| Nanocoating (PVA) | - | - | 115,126 |
| Nanocoating (Zinc Oxide) | - | - | 18,420 |
| Nanocoating (Epoxy) | - | - | 31,622 |
| Concrete for the tunnel | 74,000,000 | 28,700,000 | 21,700,000 |
| Reinforcement steel for the tunnel | 4,524,838 | 1,942,149 | 1,743,100 |
| **Total (kg)** | **186,146,406** | **32,463,940** | **24,924,974** |

Supplementary Table (B): Transportation impacts

| **Transportation Process** | **Transportation (ton-km)** | | |
| --- | --- | --- | --- |
|  | **Soil backfilling** | **EPS geofoam with Geomembrane** | **EPS geofoam with Nanocoating** |
| Dense Sand & Gravel | 4,982,480 | - | - |
| EPS geofoam | - | 61,000 | 61,000 |
| HDPE Geomembrane | - | 30,090 | - |
| Nanocoated EPS geofoam | - | - | 13,094 |
| Tunnel’s Concrete | 3,700,000 | 1,435,000 | 1,085,000 |
| Tunnel’s Reinforcement steel | 226,241 | 97,107 | 87,155 |
| **Total (ton-km)** | **8,908,721** | **1,623,197** | **1,246,249** |

Supplementary Table (C): SimaPro: quantities, processes, and database

Supplementary Table (D): Categories and parameters of ReCiPe method

| **Category** | **Impact Parameters** | **Abbreviation** | **Assessment** | **Midpoint measure** | **Endpoint measure** |
| --- | --- | --- | --- | --- | --- |
| **Human Health (HH)** | Global warming, Human health | GWP-HH | assesses the impact of greenhouse gases on human health | kg CO_2_eq | DALY |
|  | Stratospheric ozone depletion | ODP | assesses the potential of emissions to deplete the ozone layer | kg CFC11-eq |  |
|  | Ionizing radiation | IR | assesses the impact of exposure to radioactive substances on human health | kBq Co-60 to air eq |  |
|  | Ozone formation, Human health | OF-HH | assesses the effect of ozone formation on human health | kg NOx-eq |  |
|  | Fine particulate matter formation | FPMF | assesses the impact of fine particulate matter on health and the environment | kg PM2.5-eq |  |
|  | Human carcinogenic toxicity | HCT | assesses the potential of emissions to cause cancer in humans | 1,4-DCB eq. emitted to urban air |  |
|  | Human non-carcinogenic toxicity | HNCT | assesses the non-carcinogenic health effects in humans | 1,4-DCB eq. emitted to urban air |  |
|  | Water consumption, Human health | WC-HH | assesses the impact of water use on human health | m^3^ |  |
| **Ecosystem (E)** | Global warming, Terrestrial ecosystems | GWP-TE | assess the effect of emissions on terrestrial ecosystems | kg CO_2_eq | species-year” |
|  | Global warming, Freshwater ecosystems | GWP-FE | assess the impact of emissions on freshwater ecosystems | kg CO_2_eq |  |
|  | Ozone formation, Terrestrial ecosystems | OF-TE | assess the ozone formation in terrestrial ecosystems | kg NOx-eq |  |
|  | Terrestrial acidification | TA | assesses the potential contribution of emissions to acid deposition, leading to soil acidification | kg SO_2_-eq |  |
|  | Freshwater eutrophication | FWE | assesses the over-fertilization potential of water bodies due to nutrient runoff | kg P-eq. to freshwater |  |
|  | Marine eutrophication | ME | assesses the over-fertilization potential in marine environment | kg N-eq to marine water |  |
|  | Terrestrial ecotoxicity | TET | assesses the toxic substances in terrestrial ecosystems | 1,4-DCB eq. emitted to industrial soil |  |
|  | Freshwater ecotoxicity | FET | assesses the harmful substances in freshwater ecosystems | 1,4-DCB eq. emitted to freshwater |  |
|  | Marine ecotoxicity | MET | assesses the toxic effects of emissions on marine ecosystems | 1,4-DCB eq. emitted to seawater |  |
|  | Land use | LU | assesses the environmental impacts associated with land occupation and transformation | m^2^*a |  |
|  | Water consumption, Terrestrial ecosystems | WC-TE | assess the effect of water use on terrestrial ecosystems | m^3^ |  |
|  | Water consumption, Aquatic ecosystems | WC-AE | assess the impact of water use on aquatic ecosystems | m^3^ |  |
| **Resources (R)** | Mineral resource scarcity | MRS | assesses the depletion of mineral resources | kg Cu-eq | USD2013 |
|  | Fossil resource scarcity | FRS | assesses the depletion of fossil fuel | kg oil-eq |  |

Supplementary Table (E): Detailed cost breakdown for the alternatives

|  | **Unit** | **Unit Cost ($)** | **Soil Backfilling** | | **EPS Geofoam with Geomembrane** | | **EPS Geofoam with Nanocoating** | |
| --- | --- | --- | --- | --- | --- | --- | --- | --- |
|  |  |  | **Quantity** | **Total ($)** | **Quantity** | **Total ($)** | **Quantity** | **Total ($)** |
| **Materials Extraction & Manufacturing** | | | | | | | | |
| Dense sand & gravel | m^3^ | 12 | 48,800 | 585,600 | - | - | - | - |
| EPS geofoam | m^3^ | 72 | - | - | 48,800 | 3,513,600 | 48,800 | 3,513,600 |
| HDPE geomembrane | m^2^ | 5 | - | - | 323,544 | 1,617,720 | - | - |
| Bagasse | ton | 60 | - | - | - | - | 96.7 | 5,802 |
| PVA | kg | 0.6 | - | - | - | - | 115,126 | 69,076 |
| Zinc Oxide | kg | 14 | - | - | - | - | 18,420 | 257,880 |
| Epoxy | kg | 11 | - | - | - | - | 31,622 | 347,842 |
| Mixing of nanocoating | kWh | 0.03 | - | - | - | - | 863 | 24 |
| Painting of nanocoating | m^2^ | 0.7 | - | - | - | - | 292,800 | 204,960 |
| Concrete (inc. transportation) | m^3^ | 36 | 29,600 | 1,065,600 | 11,480 | 413,280 | 8,680 | 312,480 |
| Steel (inc. transportation) | ton | 860 | 4524.84 | 3,891,360 | 1,942.15 | 1,670,249 | 1,743 | 1,498,980 |
| **Total Materials Extraction and Manufacturing Cost ($)** | | | **5,542,560** | | **7,214,849** | | **6,210,644** | |
| **Transportation** | | | | | | | | |
| Dense sand & gravel | ton | 5 | 99,650 | 498,248 | - | - | - | - |
| EPS geofoam | m^3^ | 2.4 | - | - | 48,800 | 117,120 | 48,800 | 117,120 |
| HDPE geomembrane | ton | 5 | - | - | 601.79 | 3,009 | - | - |
| **Total Transportation Cost ($)** | | | **498,248** | | **120,129** | | **117,120** | |
| **Construction** | | | | | | | | |
| Soil compaction | m^3^ | 2.5 | 48,800 | 122,000 | - | - | - | - |
| EPS installation | m^3^ | 0.05 | - | - | 48,800 | 2,440 | 48,800 | 2,440 |
| HDPE welding | kWh | 0.03 | - | - | 5,693 | 158.15 | - | - |
| HDPE labor/machine | hrs | 1.5 | - | - | 540 | 810 | - | - |
| Steel fixation | ton | 5 | 4,525 | 22,625 | 1,942 | 9,710 | 1,743 | 8,715 |
| Concrete pumping | m3 | 3 | 29,600 | 88,800 | 11,480 | 34,440 | 8,680 | 26,040 |
| **Total Construction cost ($)** | | | **233,425** | | **47,558** | | **37,195** | |
| **End-of-Life** | | | | | | | | |
| Landfill | ton | 3 | 59,790 | 179,369 | 547 | 1,640 | 445 | 1,334 |
| Incinerated | kg | 0.20 | - | - | 182,179 | 36,071 | 148,187 | 29341 |
| **End-of-life Total cost ($)** | | | **179,369** | | **37,711.10** | | **30,675** | |
| **Total LCC ($)** | | | **6,453,602** | | **7,420,247** | | **6,395,727** | |
